# Editorial: Molecular Mechanisms and Treatment of MYCN-Driven Tumors

**DOI:** 10.3389/fonc.2021.803443

**Published:** 2021-11-30

**Authors:** Yusuke Suenaga, Christer Einvik, Atsushi Takatori, Yuyan Zhu

**Affiliations:** ^1^Department of Molecular Carcinogenesis, Chiba Cancer Center Research Institute, Chiba, Japan; ^2^Department of Pediatrics, Division of Child and Adolescent Health, UNN - University Hospital of North-Norway, Tromsø, Norway; ^3^Pediatric Research Group, Department of Clinical Medicine, Faculty of Health Science, The Arctic University of Norway - UiT, Tromsø, Norway; ^4^Division of Innovative Cancer Therapeutics, Chiba Cancer Center Research Institute, Chiba, Japan; ^5^Joint Laboratory of Artificial Intelligence and Precision Medicine of China Medical University and Northeastern University, Shenyang, China; ^6^Department of Urology, The First Hospital of China Medical University, Shenyang, China

**Keywords:** MYCN, neuroblastoma, brain tumor, hepatocellular carcinoma, therapeutic targets

*MYCN* encodes a transcription factor that functions as an oncoprotein, similar to other MYC family proteins, c-MYC and L-MYC. Since its discovery as an amplified gene homologous to c-*MYC* in neuroblastoma, *MYCN* has been found to be amplified and overexpressed in various tumors and promotes aggressiveness. In this special issue, we review the current understanding of the oncogenic functions of MYCN and potential therapeutic strategies against MYCN-driven tumors.

## Mechanism of MYCN Dysregulation and Drugs Targeting MYCN-Driven Tumors

The molecular mechanism of MYCN overexpression in tumors has been described in detail by R. Liu et al. Gene amplification, transcription, translation, and protein stability are involved in MYCN upregulation. It is also worth noting that the recently identified point mutation P44L in *MYCN* is an activating mutation that enhances *MYCN* transcription and protein stability.

The review by Z. Liu et al. described the structure and function of MYCN in contrast to c-MYC. It provides a detailed description of the latest findings, including the differences between MYCN and c-MYC in global transcriptional regulation, the ubiquitination and methylation mechanisms that regulate MYCN stability, and the molecular mechanisms leading to synthetic lethality in MYCN-driven tumors, including mutations in ATRX. Inhibitors that target these molecular mechanisms of MYCN expression, downstream gene regulation, or synthetic lethality are discussed in terms of their efficacy in MYCN-driven tumors. *NCYM* is an antisense gene of *MYCN* and is always amplified together with *MYCN*. Its gene product specifically stabilizes MYCN, but not c-MYC. The secondary structure of NCYM identified by Matsuo et al. may be useful for designing NCYM/MYCN-targeting drugs in the future. In addition, Takatori et al. showed that *NLRR1*, a downstream gene of *MYCN*, is also expressed in MYCN-driven tumors, including adult cancers, and that a monoclonal antibody against NLRR1 in combination with EGFR inhibitors may be an effective therapeutic strategy for these tumors. Consistent with this idea, the detailed analysis by Pan et al. of protein-protein interaction networks identified an important role for EGFR in the MYCN network and pathway, and the combination of drugs targeting MYCN or its downstream genes with EGFR inhibitors may be a promising strategy in the future. Raieli et al. comprehensively investigated the mechanisms of MYCN-induced immunosuppression and showed that the intensity of immunosuppression is associated with poor prognosis in neuroblastoma. Furthermore, they showed that BGA002, an anti-MYCN antigene peptide nucleic acid, enhances NK cell sensitivity in MYCN-amplified neuroblastomas.

## Mechanism of MYCN-Driven Tumorigenesis From an Embryological Perspective

Otte et al. explained the MYCN-driven tumorigenesis in neuroblastomas. In neuroblastoma, *MYCN* amplification is observed at the time of diagnosis, and non-*MYCN*-amplified tumors do not secondarily acquire *MYCN* amplification. In other words, unlike gene amplification in other tumors, *MYCN* amplification in neuroblastoma is a phenomenon that occurs early in carcinogenesis. Neuroblastoma development is thought to be caused by the inhibition of neural differentiation and promotion of cell proliferation in the sympathoadrenal lineages. However, recent studies revealed that overexpression of MYCN in mouse sympathoadrenal progenitors promotes neuronal differentiation without forming tumors *in vivo* and that MYCN is expressed together with CIP2A in the neural plate destined to form the central nervous system, but is excluded from the neighboring neural crest stem cell domain. Therefore, MYCN may promote neuroblastoma carcinogenesis at an earlier developmental stage than during the migration and differentiation of neural crest cells. Compared to previous models, mouse neuroblastoma developed in recently established models resembles human neuroblastoma, reflecting the gain or loss of copy number of chromosomes; however, these mouse models used Th or Dbh promoters, which overexpress MYCN relatively late in the neural crest differentiation process. To identify the origin of *MYCN*-amplified neuroblastoma, it will be necessary to investigate the effect of MYCN overexpression at an early stage of development. Explaining when and how *MYCN* gene amplification occurs during early development is an important topic that remains to be elucidated in neuroblastoma biology.

Borgenvik et al. discussed how MYCN-targeted drugs can be applied in the treatment of brain tumors, comparing the findings with those of neuroblastoma. In addition, the molecular mechanisms of subtypes, overexpression, and tumor-promoting potential of brain tumors, including the rare tumors in which MYCN is involved, are described in detail. MYCN is expressed in the hindbrain during development and is essential for cerebellar development (Shrestha et al.). Abnormalities in MYCN during cerebellar development cause medulloblastoma, one of the most prevalent causes of brain tumors in children. Consistent with the requirement of MYCN for the promotion of cell proliferation of the cerebellar granule cell precursor by SHH during development, *MYCN* is amplified and overexpressed in the SHH subtype of medulloblastoma and is involved in its aggressiveness. Amplification is also observed in Group 4 medulloblastoma, but the relationship between the physiological roles of MYCN in embryonic development and the oncogenic functions of MYCN in Group 4 medulloblastoma remains to be elucidated.

Metabolic reprogramming is an important mechanism for MYCN-driven tumorigenesis, in addition to promoting cell proliferation and inhibiting cell death. As seen in pluripotent stem cells, a function of MYCN in metabolic reprogramming is providing bio-macromolecules through the activation of the glycolytic system accompanied by upregulated mitochondrial metabolism that leads to high energy production (Otte et al.; Yoshida). By introducing the functions of downstream genes of MYCN (*ASCT2* and *GLDC*) or factors that work in concert with MYCN (ATF4 and MondoA), Yoshida explained the molecular mechanism of this metabolic reprogramming, which cannot be fully explained by the Warburg effect.

Another aspect of the stem cell-like characteristics of *MYCN*-amplified neuroblastoma is its ability to undergo symmetric division *via* inhibition of asymmetric division (Izumi et al.). MYCN promotes symmetric division by mutual regulation of the reprogramming factors OCT4 and LIN28B. In addition, a downstream gene, *HMGA1*, regulates cell fate determinants, such as NUMB, and contributes to MYCN-induced symmetric division. Because all these factors have been reported to be associated with the aggressiveness of neuroblastomas, the maintenance of symmetric division is one of the oncogenic functions of MYCN that could be targeted by drugs in the future.

## Induction of MYCN by Environmental Factors and MYCN Regulation by miRNAs

MYCN is highly expressed in hepatocellular carcinomas and correlates with cancer stem cell markers and Wnt/β-catenin signaling (Qin et al.). Indeed, it is expressed in a population of liver cancer stem cells, and high MYCN expression levels are associated with the recurrence of hepatocellular carcinoma. Unlike neuroblastoma, *MYCN* amplification is only approximately 2% in hepatocellular carcinomas, suggesting that the expression of MYCN is induced by mechanisms other than gene amplification. Hepatocellular carcinoma is not only caused by hepatitis B or C virus infection, but also by obesity-induced inflammation. Qin et al. proposed repair signaling and stress adaptation signaling in response to inflammation as the cause of MYCN induction in hepatocellular carcinomas. In addition, disruption of miRNA-mediated repression of MYCN expression, including miR-493, has attracted attention as a molecular mechanism for the high expression of MYCN in hepatocellular carcinoma. Discovery of the feedback mechanism between the miR-17-92 cluster and *MYCN* and its significance in neuroblastoma prognosis by Misiak et al. further emphasizes the importance of post-translational regulation of MYCN as a promising therapeutic target for these tumors.

This special issue focuses on the functions of MYCN and therapeutic strategies for various cancer types. It is our hope that readers will gain knowledge of cancer types outside their area of expertise, which will be useful in the development of new therapies. Elucidation of the physiological functions of MYCN, especially in developmental processes and global transcription, will also contribute to a comprehensive understanding of basic biology, not restricted to molecular oncology.

## Author Contributions

All authors listed have made a substantial, direct, and intellectual contribution to the work and approved it for publication.

## Conflict of Interest

The authors declare that the research was conducted in the absence of any commercial or financial relationships that could be construed as a potential conflict of interest.

## Publisher’s Note

All claims expressed in this article are solely those of the authors and do not necessarily represent those of their affiliated organizations, or those of the publisher, the editors and the reviewers. Any product that may be evaluated in this article, or claim that may be made by its manufacturer, is not guaranteed or endorsed by the publisher.

